# Temporary Glycosuria in Surgical Affections

**Published:** 1885-10

**Authors:** 


### TEMPORARY GLYCOSURIA IN SURGICAL AFFECTIONS.

Redard has observed temporary glycosuria in cases of fracture, in connection with simple wounds accompanied by inflammation, in inflammatory diseases of the skin and cellular tissue, in cases of erysipelas and lymphangitis, and in puerperal septicæmia. He compares these cases of glycosuria to those observed during the course of the eruptive fevers.

